# Supplementary material for: Optimization design of top beam energy absorbing member of corrugated hydraulic support with three kinds of sandwich structures based on adaptive response surface method
Source: PLoS One. 2025 May 23;20(5):e0323280. doi: 10.1371/journal.pone.0323280 (PMC12101683; doi:10.1371/journal.pone.0323280)
Supplement: Attachment — Submitted filename: Response to Reviewers01 .docx [file pone.0323280.s002.docx]

Dear editor，

Thank you for your letter and for the reviewers’ comments concerning our manuscript entitled “Optimization design of top beam energy absorbing member of corrugated hydraulic support with three kinds of sandwich structures based on adaptive response surface method” (PONE-D-24-48864). Those comments are very valuable and very helpful for revising and improving our paper, as well as the important guiding significance to our researches. We have studied comments carefully and have made correction which we hope meet with approval. In the revision, we have fully addressed the comments made by the reviewers and the editor. The completed changes are as follows:

**Reviewer #1:**

**Comment**

Due to the absence of figures in the manuscript, a thorough and proper review cannot be conducted. The figures are essential for validating the described methods and results. Please include all relevant figures and resubmit the manuscript to facilitate an effective review process.

**Response**

This issue has been corrected, thank you for your feedback .

**Reviewer #2:**

**Comment**

Suggestion:Supplementary literature review: It is recommended to provide in-depth analysis of existing impact energy absorption devices and explore the application research of corrugated structures in the field of impact energy absorption.
Improve theoretical models: It is recommended to consider factors such as material nonlinearity, geometric nonlinearity, and contact nonlinearity to establish more accurate mechanical models and conduct model validation.
Optimize simulation model: It is recommended to consider factors such as material nonlinearity, geometric nonlinearity, and contact nonlinearity, establish a more accurate simulation model, and conduct in-depth analysis of simulation results.
Improve experimental research: It is recommended to design a more rigorous experimental plan and conduct a comprehensive analysis of the experimental results.
Optimize article structure: It is recommended to optimize the article structure to make the logical relationships between chapters clearer.
Standardize language expression: It is recommended to standardize language expression to avoid the phenomenon of mixing English and Chinese.

**Response**

Thanks for your comments, it has now been revised. New references have been added to the review part for in-depth analysis of impact energy absorption devices. The application of corrugated structures in the field of impact energy absorption is the innovation point of this paper, and no similar structures have been found for impact energy absorption. For the problem of considering material nonlinearity, geometric nonlinearity, contact nonlinearity and other factors in theoretical model and optimization simulation model, our thinking is that the problem can be modeled as a linear elastic problem and solved by linear statics analysis method when material nonlinearity, geometric nonlinearity and contact nonlinearity are not considered. This method does not involve nonlinear effects, and can be numerically solved and verified by conventional linear finite element method (such as elastic mechanics analysis). It is suitable for mechanical analysis and optimization simulation when the strain is small, the material is approximately linear, and the contact is not complicated, so the nonlinear problem is not considered. The experimental scheme has been re-elaborated, and the analysis of experimental results is based on experiments. The structure of the article has been adjusted, the order of the original chapter 3 and chapter 4 has been switched, and the logical expression of the structure has been basically smooth. For the mixed use of English and Chinese has been modified, thank you for your correction.

**Reviewer #3:**

**Comment 1**

The discussion on the mechanical properties of these type of tubes does not lead to conclusions regarding the advantages and disadvantages of using these structures in engineering applications. The author needs to add some new contents in the Abstract and Conclusion to better illustrate the innovation and the application of this research.

**Response 1**

This issue has been corrected, thank you for your feedback .

**Comment 2**

The illustrations of the manuscript do not appear to be attached, please find out why.

**Response 2**

This issue has been corrected, thank you for your feedback .

**Comment 3**

The grammar of the whole manuscript should be checked

**Response 3**

This issue has been corrected, thank you for your feedback .

**Reviewer #4:**

**Comment 1**

The paper is not completed.

**Response 1**

This issue has been corrected, thank you for your feedback .

**Comment 2**

Some figures are not attached.

**Response 2**

This issue has been corrected, thank you for your feedback .

**Comment 3**

The literature review also not present clearly and almost not included.

**Response 3**

This issue has been corrected, thank you for your feedback .

**Comment 4**

The formatting of the paper also poor.

**Response 4**

This issue has been corrected, thank you for your feedback .

All authors have read and approved the re-submission of the manuscript! If you have any questions, please let me know!

We are looking forward to hearing from you!

Sincerely yours,

Qian Liu
