# Supplementary material for: Optimization design of top beam energy absorbing member of corrugated hydraulic support with three kinds of sandwich structures based on adaptive response surface method
Source: PLoS One. 2025 May 23;20(5):e0323280. doi: 10.1371/journal.pone.0323280 (PMC12101683; doi:10.1371/journal.pone.0323280)
Supplement: Attachment — Submitted filename: Comments.docx [file pone.0323280.s001.docx]

Overview and general recommendations:

This is an article about the application of corrugated structure in impact absorbing advanced support hydraulic pillar. According to the national design standard for advanced support hydraulic pillars, the corrugated energy-absorbing components of the support beams are optimised and their cushioning and energy-absorbing characteristics are investigated. The manuscript is interesting and the reviewer recommends acceptance with modifications. Specifically, the following points should be noted for improvement:

1. The expression of several proper nouns in the manuscript is inconsistent, so please check it carefully. For example, the expressions related to the three energy-absorbing components are inconsistent.

2. In subsection 1.2, it is mentioned that ‘When the reaction force exceeds the initial peak crushing load and after hydraulic support safety valve failures, the deformation displacement is considered effective if the reaction force is less than 1.3 times the initial peak crushing load.’ What is the basis for this, please include necessary explanations or references.

3.The introductory part deals with only about 20% of the references in the last three years, which should be increased appropriately to enrich the study. (Recommended as follows: https://doi.org/10.1016/j.tws.2024.112415

https://doi.org/10.1007/s43452-024-00895-9

https://doi.org/10.1016/j.engfracmech.2024.110009)

4. Figures 3.2 and 3.3 seem to lack the necessary textual descriptions in the manuscript; moreover, the titles of the figures are inconsistent with the contents of the figures (e.g., energy absorption vs. specific energy absorption in Fig. 3.2; relative energy absorption vs. specific energy absorption in Fig. 3.2).

5. The curves in Figure 4.8 are interesting and, unlike the other two structures, the reader may expect a more in-depth analysis.
